# A comprehensive survey of permethrin resistance in human head louse populations from northwest Iran: ex vivo and molecular monitoring of knockdown resistance alleles

**DOI:** 10.1186/s13071-023-05652-0

**Published:** 2023-02-06

**Authors:** Mohammad Bagher Ghavami, Sanaz Panahi, Seyede Maede Nabati, Maryam Ghanbari, Behrooz Taghiloo

**Affiliations:** Department of Medical Entomology and Vector Control, School of Medicine, Zanjan University of Medical Sciences, Zanjan, Iran

**Keywords:** *Pediculus humanus capitis*, Head louse, Biological assay, Pyrethroid resistance, Voltage-sensitive sodium channel mutations, Mitochondrial cytochrome* b* groups, Knockdown resistance genotyping

## Abstract

**Background:**

Head louse infestation is an important public health problem, and expanding resistance to permethrin is a major challenge to its control. The mapping and detection of pyrethroid resistance are essential to the development of appropriate treatments and ensure the effectiveness of current measures. The aim of this study was to present the phenotypic and genotypic basis of permethrin resistance and identify knockdown resistance (*kdr*) mutations in head louse populations in northwestern Iran.

**Methods:**

Adult head lice were collected from 1059 infested girls in Ardebil, East Azerbaijan, West Azerbaijan and Zanjan Provinces, northwestern Iran. The toxicity of permethrin and the possible synergistic effect of piperonyl butoxide (PBO) on this toxicity were assessed using bioassays. Fragments of voltage-sensitive sodium channels (*vssc*) and cytochrome b (*cytb*) genes were amplified and analyzed for the detection of knockdown resistance (*kdr*) mutations and mitochondrial groups. Moreover, genotypes of the two hot spot regions of the *vssc* gene were determined by melting curve analysis of amplicons.

**Results:**

A total of 1450 adult head lice were collected during 2016–2021. Live lice were exposed to a dose of 1% permethrin for 12 h, and the median lethal time (LT_50_) and time to achieve 90% mortality (LT_90_) were determined to be 6 and 14.8 h, respectively. Topical application of 2 and 16 ng permethrin per louse resulted in 25% and 42.11% mortality, respectively. Pre-exposure of samples to 3% piperonyl butoxide had no synergistic effect on the effects of permethrin. Analysis of the 774-bp *vssc* gene fragment showed the presence of the M815I, T917I and L920F mutations, wild-type and T917I mutation, in 91.6%, 4.2% and 4.2% of samples, respectively. Investigation of the mitochondrial *cytb* gene demonstrated the predominance of clade B. The frequency of domain II segment 4 (S4)-S5 *kdr* genotypes in mitochondrial groups was identical, and heterozygotes were present in 93.5% of samples. A significant difference was detected in the frequency of domain IIS1-S3 *kdr* genotypes, and the frequency of resistant alleles and heterozygotes was higher in clade B than in clade A.

**Conclusions:**

The presence of *kdr* mutations in the *vssc* gene and the non-synergist effect of PBO indicate that pyrethroid target site insensitivity is the main resistance mechanism. This phenomenon and the high frequency of resistant alleles necessitate that new pediculosis management programs be developed. Further studies need to be conducted to identify all factors contributing this resistance and to develop alternative pediculicides.

**Graphical Abstract:**

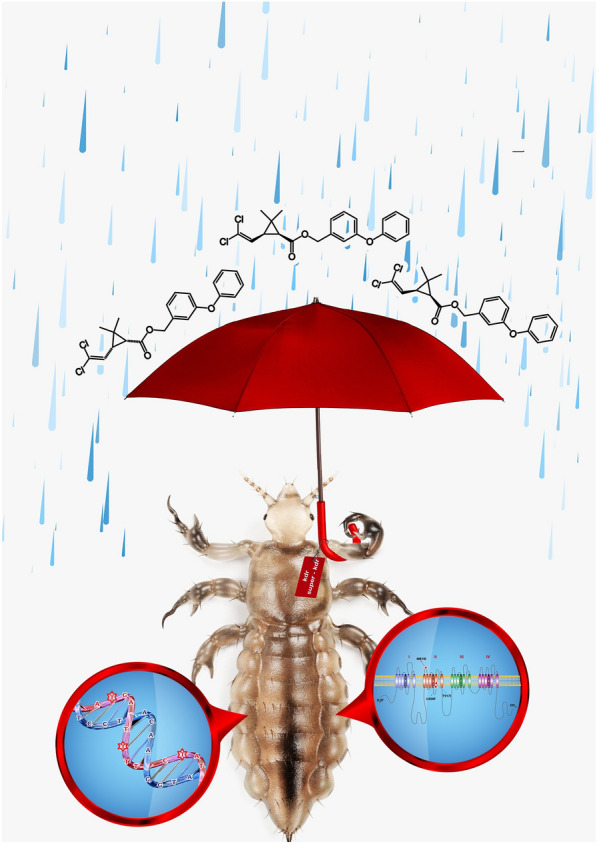

## Background

The human head louse, *Pediculus humanus capitis* De Geer, 1767, (Anoplura: Pediculidae), is an obligatory hematophagous ectoparasite that is associated with several deleterious public health threats worldwide [1]. Infestation with this louse, referred to as Pediculosis capitis, has increased among people in recent years, particularly in children aged 3–11 years [2, 3]. This condition induces skin irritation, superinfection from scratching and psychological distress, and also results in the social isolation of affected individuals and exclusion from school [4, 5]. On the basis of the variable morbidity, social stigma and decreased efficiency associated with the louse burden, the WHO has newly added pediculosis to the priority list of neglected tropical diseases [6]. The different forms of head lice are not typically considered to be major vectors of human disease agents and therefore do not raise public health concerns. However, some ecotypes of this louse, such as body lice, can transmit several life-threatening pathogens [7–11]. Head lice are often perceived by the public as an embarrassing social nuisance that requires particular treatment, demanding potentially toxic pediculicides and home remedies [12].

Over the past 80 years, the control of pediculosis has largely been dependent upon the availability of natural and synthetic insecticides, starting with dichlorodiphenyltrichloroethane (DDT; in 1943), natural pyrethrins (1945), the organochlorine (OC) lindane (1960), organophosphorus insecticides (malathion; 1971), carbamates (carbaryl; 1977) and synthetic pyrethroids (permethrin and phenothrin; 1992). Other control options include topical non-neurotoxic agents (e.g. dimeticone), topical plant-based compounds or essential oils and physical methods (e.g. bug busting), albeit with variable results. Currently, oral treatment with ivermectin is not recommended as a first-line treatment but this product might be useful for head louse infestation unresponsive to topical insecticide treatment [12–15].

Permethrin, commonly used as an over-the-counter chemical pediculicide, is now suggested as the first-line treatment for pediculosis capitis [14–17]. This compound, DDT and natural pyrethrins share a common target site in the nervous system, namely the voltage-sensitive sodium channel (VSSC) proteins, and cause neural excitation by increasing persistent sodium current, resulting in nerve depolarization and hyperexcitation, followed by neuromuscular paralysis (knockdown) and death [18]. Exclusive use of this compound as well as self-treatment with other products, especially those containing pyrethrins, share a common mechanism of action on VSSC in the nervous system of insects. Moreover, their sole and continued use has placed a high selective pressure on head louse populations, which has led to the emergence and spread of resistance to pyrethroids in many parts of the world. This condition may cause treatment failures and result in chronic infestations, ultimately requiring additional episodic treatments. The development of resistance to pyrethroids also generates economic costs, potential toxicity, anxiety, inconvenience and a further increase in drug pressure [19].

Insecticide resistance is particularly problematic in the control of human lice for several reasons: (i) human lice are obligate human blood feeders exposed to pediculicides at all life-stages; (ii) they have short generation time and high fecundity; and (iii) there are only a few pediculicidal products, the majority of which share common chemistry and elicit cross-resistance. Because of these issues, louse resistance to most commercial pediculicides has occurred and is escalating [13, 20].

To date, numerous techniques have been reported for detecting resistance in head louse populations, but toxicity bioassays and molecular methods are the conventional approaches for monitoring and expressing resistance. Bioassay strategies are often unfeasible in routine monitoring as they need a sufficient number of live specimens, and obtaining a sufficiently large number is difficult. Furthermore, monitoring by these methods can be compromised, and the resistance alleles are recessive [21]. Molecular assays have advantages, such as their requirement for a smaller sample size and ability to accurately identify the gene(s) that is/are associated with the resistance mechanisms. However, sophisticated and costly equipment and reagents are required to perform molecular assays, which may not be readily available in developing countries [19, 20, 22, 23].

Molecular analyses of susceptible and resistant strains of head lice have revealed that there are a number of point mutations in the *vssc* gene, suggesting that the substitution of amino acids is the main reason for enhancing resistance to pyrethroids [19, 20, 22, 24]. Such alterations in the nerve cell membrane mitigate the effect of the insecticide on the nervous system, and leading to paralysis or knockdown resistance (*kdr*). This intrinsic trait, which is related to DDT and pyrethroids, was first identified in the housefly, *Musca domestica,* and has been documented in several different species of agricultural and public pests [25, 26].

In head lice, *kdr*-type resistance to pyrethroids is mainly associated with three-point mutations (amino acid substitutions on the M815, T917 and L920 residues) in two hot spot areas, encoding the domain II segment 1 (S1)−S5 region located on the α-subunit of the VSSC pyrethroid-receptor site. The first area includes the main *kdr* mutation site, namely M815, encoding the domain II S1 (IIS1) region, and the second area contains two putative *kdr* mutation sites, especially T917 and L920 residues, encoding the domain II S5 (IIS5) region. In head lice, the M815I and L920F mutations are always found together with the T917I mutation and these probably play a synergistic role in enhancing pyrethroid resistance [19, 20, 22, 23, 27]. Further experiments using site-directed mutagenesis at the corresponding amino acid sequence positions of the house fly para-orthologous VSSC *a*-subunit (*Vssc1WT*) gene and heterologous co-expression with the sodium channel auxiliary subunit of house fly (*Vssc*b) in *Xenopus* oocytes showed that the T917I mutation was the main cause of permethrin resistance in head lice via a *kdr*-type nerve insensitivity mechanism [27]. Previous investigations have also identified some novel mutations (H813P, L899V and D953G) in the IIS1-2 and five sequential mutations (I927F, L928A, R929V, L930M, and L932M) in the IIS5 of the *vssc* gene [28, 29]. However, the function of these mutations in pyrethroid resistance is unclear.

Various molecular techniques, including Sanger sequencing of PCR products, quantitative multiplex sequencing [30–32], PCR-restriction fragment length polymorphism (PCR–RFLP) [33], real-time PCR amplification of specific allele (rtPASA) and serial invasive signal amplification reaction (SISAR) [34–36], have been applied to identify resistant head lice in previous studies. Also, melting curve analysis genotyping coupled with quantitative PCR fluorescent resonance energy transfer technology (FRET) [37, 38] has been used to identify head lice carrying resistance. Each of these methods has its own unique merits and demerits, as well as varying requirements for detection instruments, technical skills and associated costs of the reagents. Nonetheless, some of these methods are time-consuming and impractical for use in large-scale investigations.

Recent studies have indicated that among Asian countries, Turkey and Iran present a high rate of pediculosis capitis, with reported prevalence varying from 0.3% to 42.6% [39–41]. A remarkably high prevalence of head lice among females has also been recorded in the northwest of Iran [42–44] where two clades (A and B) of head lice have been reported, with clade B being the dominant group in all regions. The distribution pattern of clade A depends on socio-economic, biological, operational and technical conditions [45]. For the past three decades, the application of permethrin in the form of shampoo has been the first line of treatment for pediculosis, with gamma-hexachlorocyclohexane, dimeticone and phenothrin used as alternatives to permethrin. Thus, in terms of the selective pressure of the permethrin insecticide, there is a possibility of resistance in these populations of this ectoparasite. Despite extensive surveys on the epidemiology of pediculosis, there are very few studies on the expression of resistance status and susceptibility levels of different populations of head lice to common insecticides in Iran. Resistance of the body louse to gamma-hexachlorocyclohexane has been reported from Iran following the introduction of OC in the 1960s [46]. Several mutations have also been detected upstream and downstream of the M815I, T917I and L920F mutations by monitoring different head louse populations in the country [28, 29, 47]. While the role of these mutations in resistance is obscure, their introduction could challenge the expression of genetic groups in head louse populations.

Given the frequent control failures in the pyrethroid treatment of head louse infestations, there is a pressing need for information on the extent of pyrethroid resistance and mechanisms of resistance, as well as for a highly effective strategy to monitor populations for resistance and the identification of suitable replacements or alternatives for current pediculicides. The objectives of the present study were to investigate the level of permethrin susceptibility within different head louse populations and to assess the distribution of resistance-associated alleles. To address these aims, we examined permethrin resistance in head louse populations from northwestern Iran. By means of PCR amplification and sequence analysis of the para-orthologous gene fragments of the sodium channel, we identified point mutations associated with permethrin resistance. Herein, we report rapid and reliable methods for monitoring mitochondrial A and B clades in human head lice. In this work, we utilized double real-time PCR (RT-PCR) as a high-throughput population genotyping method to determine the *kdr*-type mutation frequency at each allele. It is envisioned that such methods will be used as the initial resistance monitoring tool for the routine detection of permethrin resistance in field populations.

## Methods

### Collection of head lice

Adult head lice were collected from infested girls in Tabriz, Mianeh, Bostanabad, Varzeghan (West Azerbaijan Province), Tekab, Salmas and Khoy districts (East Azerbaijan Province), Zanjan, Mahneshan and Khodabandeh districts (Zanjan Province) and Khalkhal and Ardabil districts (Ardabil Province), northwestern Iran, from 2016 through 2020. The most common treatment for pediculosis in these areas has been the use of 1% permethrin in shampoos for the last three decades.

In this study, volunteers wore a disposable white apron, their hair was combed with a metal head lice comb and the collected lice were transferred to 15-ml Falcon tubes for bioassay tests. For the molecular study, samples of lice from each infested individual were transferred to a 1.8-ml cryovial containing 70% ethanol. All samples were kept at − 20 °C for further analysis.

### Bioassays

Technical-grade 92% permethrin (25%* cis*, 75%* trans*) was obtained from Heranba Industries (Mumbai, India), and 90% piperonyl butoxide (PBO) was obtained from Otto Chemie Pvt. Ltd. (Mumbai, India).

Stock solutions of 3% permethrin and 9% PBO, used for paper impregnation, and of 6% and 48% permethrin, respectively, used for topical application, were prepared as volume/volume solutions in silicone fluid. The working solution of these preparations was mixed 2:1 with trichloroethylene to obtain the desired test concentration. Filter papers (Whatman no. 1, diameter: 8 cm; Whatman plc, Maidstone, UK) were impregnated with 0.5 ml of the working solution, left to dry in a fume hood and then wrapped in aluminum foil and stored at − 20 °C until used.

A standard method for ex vivo assessment of lice susceptibility, the contact assay, was used as described previously [22, 48–55]. Briefly, the impregnated papers were placed at the bottom of an 8-cm Petri dish, and a group of 10–15 lice was exposed to treated disks. The knockdown and mortality rates were then assessed after 15, 30, 45 and 60 min and 2, 4, 8, 12 and 16 h of exposure. In the tests on the effect of pre-exposure of specimens to PBO, lice were exposed for 1 h to impregnated papers, then transferred to a clean recipient Petri dish and maintained for 30 min to prepare for the test.

In the topical application studies, 0.1 μl of working solution was placed on the dorsal abdomen of each louse using a 5-μl Hamilton syringe [53, 56, 57]. Control insects received only 0.1 μl of trichloroethylene-silicon solution. Topical application was conducted under a stereomicroscope to ensure that each drop was placed correctly onto the specimen's abdomen. Following the treatment, the lice were placed on a clean filter paper disk fitted into a Petri dish and then kept at 18 ± 0.5 °C and 70–80% relative humidity in the darkness; mortality was assessed at 18 h after topical application. The lice were then examined under a stereomicroscope, and their survival was evaluated based on predefined criteria with regard to activity, ataxic signs and gut and leg movements. Lice were judged to be dead if they had no vital signs even after stimulation with forceps.

The bioassay data were assessed by probit analysis using PreProbit version 1.6.3 software [58]. Lethal time (LT) values were estimated when the Chi-square (*χ*^2^) value was small (*P* > 0.10). Confidence limits were calculated at *t* = 1.96 when the *χ*^2^ value was high (*P* < 0.10); heterogeneity was suspected and* t* for confidence limits was chosen based on the heterogeneity factor. Log time versus logit percent mortality regression analysis was generated to determine LT_50_ values (time to 50% mortality; median mortality), while the maximum log-likelihood ratio test was performed to examine the equality of the slope and intercept of the regression lines. A comparison of bioassay data was visualized in graphic form by plotting exposure time (logit) versus percentage mortality (probit). The levels of PBO synergism were estimated by the synergistic ratio (SR), i.e. the normalized LT_50_ of permethrin-treated lice divided by that of the PBO-permethrin-treated lice.

### Molecular studies

#### Genomic DNA extraction

Genomic DNA (gDNA) was extracted from samples as described previously [45, 59] with some modifications. In brief, lice were rinsed 3 times with sterile distilled water, dried on a sterile filter paper and stored at − 70 °C for 2 h. The frozen lice were placed individually in a sterile glass Petri dish and cut into small pieces in 500-µl TENS lysis buffer (100 mM of Tris HCl [pH 8.0], 10 mM EDTA, 0.5 mM NaCl, 1% W/V SDS). Each mixture was then transferred to 1.5-ml tubes and homogenized with glass beads by an electric homogenizer for 2 min, following which 20 µg of proteinase K was added to the homogenates and the homogenates incubated at 55 °C for 3 h. After incubation, the samples were stored at 65 °C for 10 min, then 100 µl of 8 M potassium acetate was added to each tube; the tube was mixed gently and kept in an ice bath for 30 min. The samples were then spun at 1000 *g* for 6 min, and the supernatants were transferred to fresh tubes. A 300-µl aliquot of ice-cold absolute ethanol was then added to each tube, followed by centrifugation at 5000 *g* for 10 min. The pellets were washed in 0.2 ml of 70% ethanol before a second centrifugation at 5000 *g* for 10 min. The final pellet was dried at room temperature and re-suspended in 50 µl of TE buffer (SinaClon, Tehran, Iran).

#### Nested PCR amplification of the mitochondrial cytochrome *b* gene

The 450-bp fragment of the mitochondrial cytochrome* b* gene (*cytb*) was amplified as described previously [45]. Nested amplification of *cytb* fragments was set up in a final reaction volume of 25 µl, containing 1 µl (approx. 20 ng) of DNA mass of PCR product, 8 pM of each outer primer (PBF and PBR), 10 pM of each inner primer (FCB: 5′-ATTCTCCTCCACCAACAC-3′; RCA: 5′-AACAAATCCCAACAAGATAAA-3′) and 12.5 µl of 2× Red Master Mix (Ampliqon, Odense, Denmark). The PBF and PBR primers (outer primers) were used as previously described [45]. The inner primers (FCB and RCA) were designed from the highly conserved region of the *cytb* gene in reference clade A and B sequences (EU93439, AY695942, AY696013 and AY696047). The PCR cycling program consisted of one cycle of initial denaturation at 95 °C for 5 min, followed by 30 cycles of 30 s at 95 °C, 30 s at 52 °C and 30 s at 72 °C for 30 s, with a final extension step at 72 °C for 7 min.

#### Allele-specific amplification of the voltage-sensitive sodium channel gene

Using the specific primers PCAF (forward: 5′-TGTGGCCTTACTTGTATTCGAC-3′) and PCBR (reverse: 5′CATTGTCAGCGGTGGGAGCAGA 3′) [35], we performed an initial PCR to obtain a fragment of the voltage-sensitive sodium channel gene (*vssc*). The reverse primer of the conventional PCR, PCAR (5′- ATCTGGGAAGTTCTTTATCCA-3′), was designed by sequencing and nucleotide alignment of the initial PCR products. Amplification of the α-subunit of the *vssc* gene was performed in a total reaction volume of 25 ml, containing 10–20 ng of gDNA, 1 pM of each primer and 12.5 µl of 2× Red Master Mix (Ampliqon). The PCR cycling program consisted of one cycle of initial denaturation at 95 °C for 5 min, followed by 35 cycles at 95 °C for 30 s, 54 °C for 60 s and 72 °C for 1 min, with a final extension step at 72 °C for 10 min.

#### DNA sequencing and analysis of PCR products

A 5-µl sample of each PCR product was assayed by electrophoresis in a 1.5% agarose gel at 80 V for 45 min and the products visualized under a UV light. To monitor the putative *kdr* mutations in the *vssc* gene, some of the amplicons were selected randomly, purified by PCR Clean-Up Kit (SinaClon) and sequenced bi-directionally by Macrogen (Seoul, Korea) with the forward and reverse specific primers. The nucleotide sequences of samples were aligned by Clustal Omega (https://www.ebi.ac.uk/Tools/msa/clustalo/) and analyzed by the aid of BioEdit software [60]. The *kdr* mutations were confirmed by comparing the amino acid sequences of samples with reference *P. humanus capitis* alignments (GenBank accession no. AY191156). The nucleotide sequences in some of the samples were submitted to the NCBI GenBank (see Results).

#### SYBR Green RT-PCR assay

Two single-plex RT-PCR assays were developed to detect the pyrethroid resistance genotype in domain IIS1-S5 of the *vssc* gene. The first assay targeted the mutation in domain IIS1-S3, and the primers were designed to flank the upstream and downstream of the M815I mutation. Reaction with the forward PCAF and reverse RPCT (5′-CCAGTCCAAGTTCCAATAATGAC-3’) primers yielded a 357-bp amplicon. The second assay targeted the domain IIS4-S5 mutations, T917I and L920F, and primers used in the reaction were FPRT (5′-GTCATTATTGGAACTTGGACTGG-3′) and PCAR, which produced a 439-bp amplicon. Each reaction (20 μl) contained 4 μl of sample gDNA (approx. 40 ng), 900 nM of each primer and 10 μl of Ampliqon SYBR Green Master Mix High Rox (Ampliqon). The reference mutant (samples with M815I, T917I and L920F mutations), wild-type head louse (samples without deduced mutations) and ddH2O were used as the positive control of the resistant alleles, positive control of the susceptible alleles and negative control, respectively. The PCR reaction was started with a 95 °C hold step for 3 min, followed by 40 cycles at 95 °C for 30 s, 58 °C for 45 s and 72 °C for 45 s, with a final extension step of 72 °C for 10 min and a last hold step for 30 s at 90 °C. Melt curve analysis was initiated from 58 °C to 95 °C at a rate of 0.1 °C/s, and the change in the fluorescence of SYBR Green was measured continuously on the green channel of the ABI StepOnePlus RT-PCR system (Applied Biosystems, Thermo Fisher Scientific, Waltham, MA, USA). Melt curves were generated in the StepOne 2.3 software (www.lifetechnologies.com). Gain optimization of all tubes was carried out before the melt analysis to obtain high-quality melting peaks. Each specimen was named as resistant (RR), susceptible (SS) and heterozygous (RS) according to the melt curves.

## Results

**Fig. 1:**
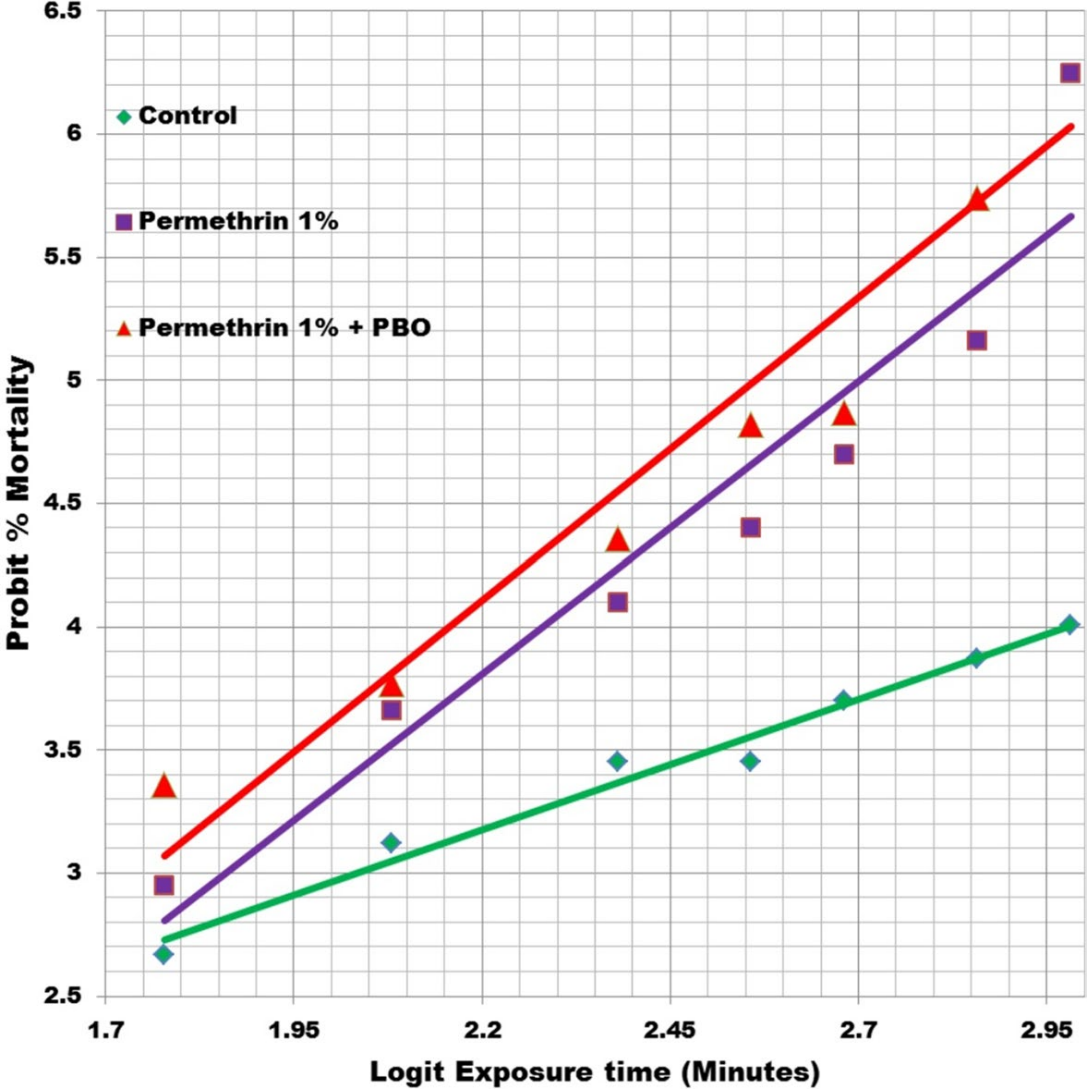
Mortality responses of head louse (*Pediculus humanus capitis*) samples exposed to 1% permethrin and pre-exposed with synergistic 3% piperonyl butoxide. PBO, piperonyl butoxide

A total of 1450 adult head lice were collected from 1059 infested girls in the studied areas. Almost 385 lice were exposed to 1% permethrin in groups of 10–12 lice using the continuous contact method. An exposure duration of 12 h seemed to be sufficient for assessing mortality based on observations that about 85% of untreated lice survived this length of time. Overall, 1% permethrin exhibited low efficacy: at 60 min after exposure, 95.97% (191/199) of lice were alive; after 120 min, 90% (182/199) of the lice were still actively moving; and at 480 min, only 38.24% (88/199) were dead (Fig. 1). The susceptibility values, LT_50_, LT_90_ (lethal time for 90% mortality) and slope of probit regression of the permethrin-treated group are shown in Table 1. The slope of the dose–response regression line did not significantly decrease from 2.78 (*χ*^2^ = 10.33, *P* = 0.00). For LT_50_ and LT_90_, the values were recorded at 354 and 891 min after exposure, respectively.

**Table 1:** Probit analysis of permethrin mortality and synergistic effect of piperonyl butoxide in head louse samples of northwest Iran

Study group	Sample size	a^a^	b^b^	*χ*^2^	*P*	LT_90_ (95% CI)
Permethrin	199	− 7.46	2.78	10.33	0.00	22.5 (14.8–95.2)
Permethrin + PBO	186	− 6.64	2.55	10.71	0.00	20.9 (13.5–55.2)

* CI* Confidence interval,* LT*_*50*_ median lethal time (h),* LT*_*90*_ time (h) at which 90% of samples had died, *PBO* piperonyl butoxide

^a^Intercept of the regression line

^b^Slope of the regression line

The ex vivo treatment of PBO with permethrin was carried out on 186 head lice. After 60 min of exposure, only 7% (13/186) of the lice were dead; a mortality rate of 80.11% was achieved at 720 min (Fig. 1). The permethrin mortality responses of the lice samples treated with the synergistic PBO and permethrin were not statistically different from those of the groups not treated with the synergistic PBO and permethrin (*χ*^2^ = 2.96, *df* = 6, *P* = 0.81). The LT_50_ and LT_90_ values and the slope of the dose–response regression line in the PBO pre-exposed group were in the range of the group which had not been pre-treated with PBO (Table 2). A comparison of LT_50_ values of these groups resulted in an SR value of 1.17, indicating that PBO had no synergistic effect on permethrin.

**Table 2:** Distribution of knockdown resistance-like M815 alleles of voltage-sensitive sodium channel gene (*vssc*) in mitochondrial groups of head louse populations

Lice group	No. of lice	Genotype^a^			Frequency of resistance allele (%)	H–W (*χ*^2^)^b^	*P*^c^	*F*_is_^d^
		*R*/*R*	*R*/*S*	*S*/*S*				
Clade A	36	12 (33.33)	11 (30.55)	13 (36.12)	48.61	5.43	0.06	0.39
Clade B	260	55 (21.15)	190 (73.08)	15 (5.77)	57.69	64.21	0	− 0.49
Total	296	67 (22.63)	201 (67.9)	28 (9.47)	56.59	43.21	0	− 0.38

^a^*S* and *R* are susceptible and resistant alleles, respectively. The percentages of each genotype proportion is shown in parentheses

^b^Populations were tested for the Hardy–Weinberg equilibrium using a Chi-square test

^c^Probability (*P*) values of the Chi-square test

^d^*F*_is_, inbreeding coefficient) = 1 — (observed frequency of heterozygotes/expected frequency of heterozygotes). *F*_is_ values > 0 indicate heterozygote deficiency and positive inbreeding; *F*_is_ values < 0 indicate heterozygote excess and negative inbreeding

**Fig. 2:**
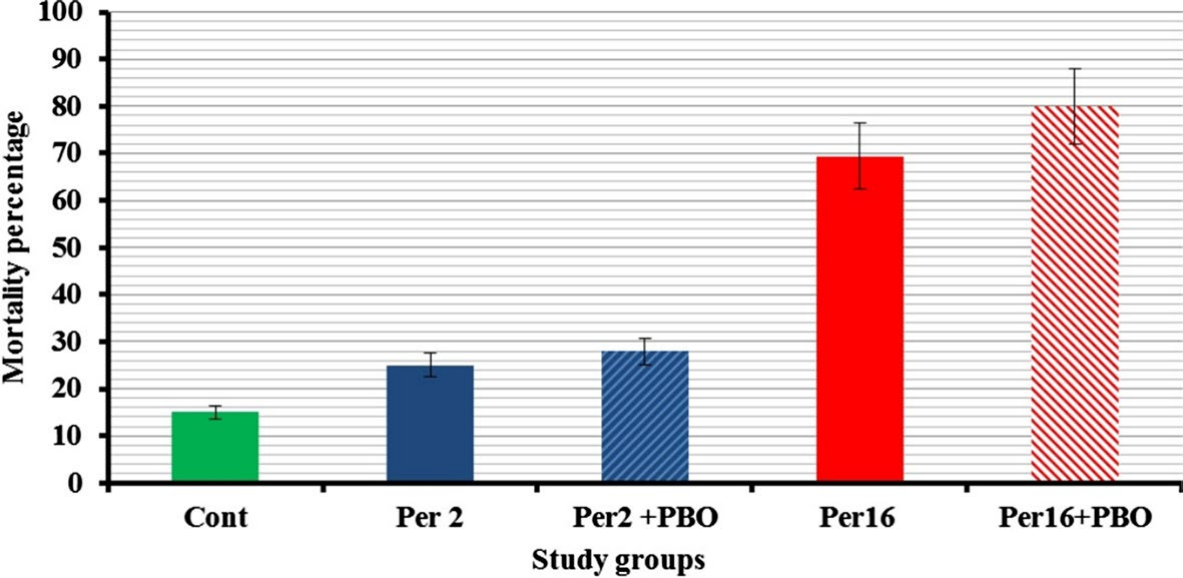
Mortality responses of head louse samples to topical application of permethrin and the synergist PBO. Cont, Control; Per 2, dose of 2 ng permethrin per louse; Per2 + PBO, treatment with 2 ng permethrin per louse plus synergist PBO; Per16, treatment with dose of 16 ng permethrin per louse; Per16+PBO, treatment with dose of 16 ng permethrin per louse plus synergist PBO

In the tests with topical application of permethrin, 80 samples of head louse were tested with the diagnostic dose of 2 ng per head louse and with a dose eightfold higher than the diagnostic dose (16 ng/ insect). The mortality responses were < 80% in these two applied doses of permethrin (Fig. 2). Application of 2 and 16 ng of permethrin per insect caused 25.0% (8/32; 95% confidence interval [CI]: 13.25–42.11%) and 69.44% (25/36; 95% CI: 53.14–82.0%) mortality in the studied samples, respectively. The mortality values of these groups following pre-exposure with the synergist PBO were 31.25% (10/32; 95% CI: 17.95–48.57%) and 80.0% (28/35; 95% CI: 64.11–89.96%), respectively. The mortality responses of samples treated with PBO and permethrin were not statistically different from the samples not exposed to PBO at the studied doses of 2 and 16 ng of permethrin per insect (*χ*^2^ < 0.7, *P* > 0.78).

Initial PCR amplification of the *vssc* gene fragment was conducted in 30 randomly selected samples, and five amplicons were sequenced. These sequences were 999 bp in size, and multiple alignments showed that they were completely conserved. The nucleotide sequence of a representative sample was submitted to GenBank with the accession number OM891554. The BLAST search of the deposited sequence shared 99% nucleotide similarity with the *P. humanus capitis vssc* gene (MZ688383, AY191157, AF542064 and DQ062568).

**Fig. 3:**
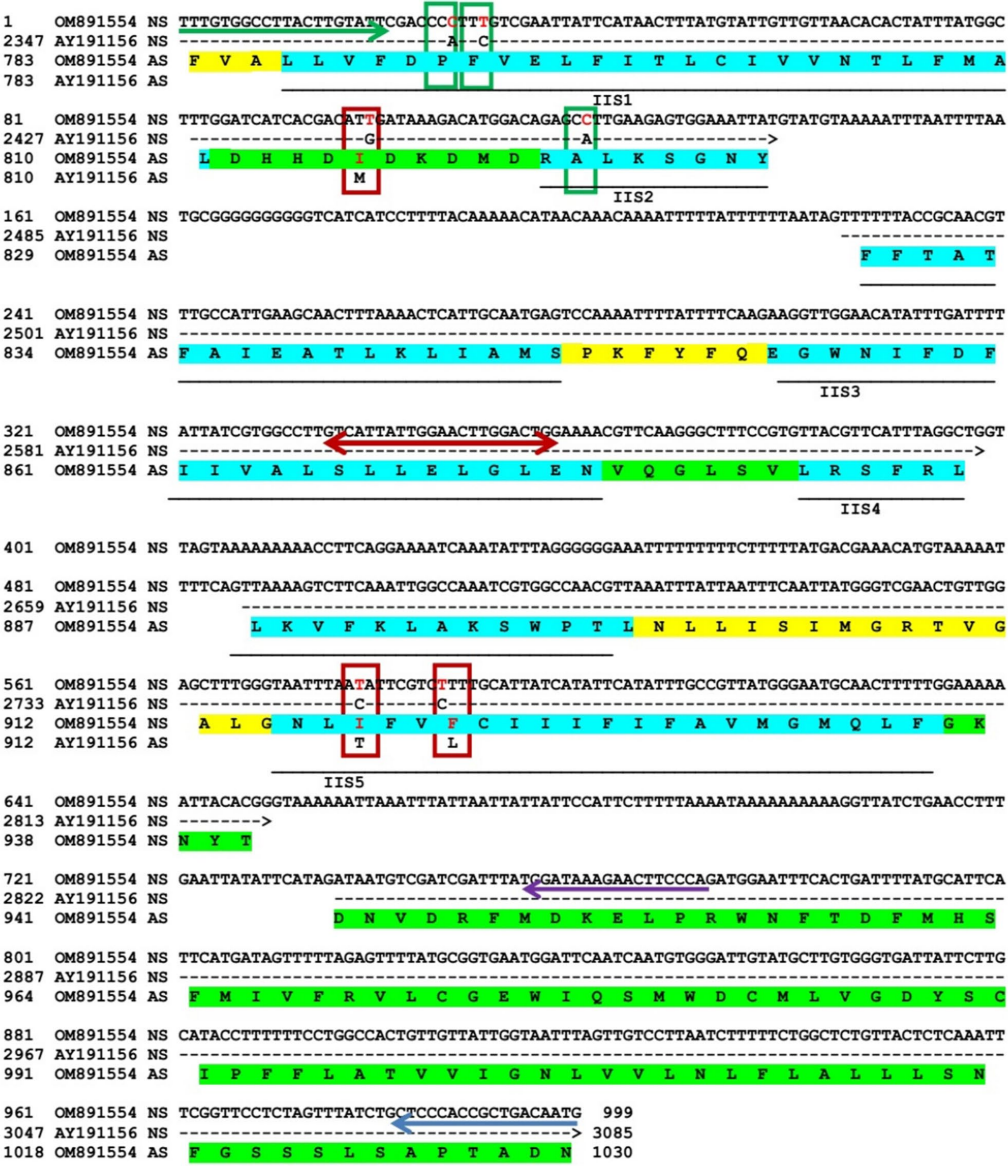
Alignment of the domain IIS1–S5 fragment of the voltage-gated sodium channel gene (*vgsc*) of the head louse *Pediculus humanus capitis* with accession no. OM891554 against the reference *vssc* gene of wild-type *P. humanus capitis* (accession no. AY191157). The mutation sites are indicated in differently colored vertical boxes: red depicts the nonsense mutation at M815, T917 and L920 sites; green depicts silent mutations with transversion (at P790 and A822 sites) and transition (at F791 site). Nucleotides (nt) 1–138 include exon I, nt 139–224 include intron I, nt 225–398 include exon II, nt 399–486 include intron II, nt 487–649 include exon III, nt 650–735 include intron II and nt 736–999 include exon IV. NS and AS represent nucleotide and deduced amino acid sequences, respectively. The locations of reverse primers are presented in blue (initial PCR), violet (conventional PCR) and red (SYBR Green real-time PCR) highlights, and the forward primer is marked with a green arrow. The segments, intracellular intersegment linkers and extracellular intersegment linkers of VSSC domain II are highlighted with blue, yellow, and green, respectively

The amplified fragment of the initial PCR contained three introns (I, 86 bp; II, 88 bp; III, 86 bp) and four exons (3ʹ, 138 bp; internal I, 174 bp; internal II, 163 bp; 5ʹ, 264 bp), and encoded 248 amino acids (Fig. 1). Analysis of the representative sequence against the reference strain of susceptible head lice (AY191157) showed 99.19% (732/738) identity in the exon section, with a difference in six nucleotides, four of which were in exon 3′ and the two others were in internal II exon (Fig. 3). One nucleotide mismatch of exon 3′, substation at position 99 (G/T), was a nonsense substitution that led to the mutation of M815I in the amino acid sequence. The other substations of exon 3′, transversions at positions 27(A/C) and 120 (A/C) and transitions at positions 30 (T/C), i.e. the P790, A822 and F791 mutations, were silent; these mutations were in the P790, A822, and F791 sites, respectively. Despite several variations in exon 3′, two *kdr* known mutations, substations of I917 (isoleucine/I: AAT) and F920 (phenylalanine/F: TCT) with T917 (threonine/T: ACA) and L920 (leucine/L: CTT) amino acids, were detected in internal exon II, respectively.

Conventional PCR was performed in 60 randomly selected samples using the FPKD and R2PKD primers. Amplified products were subjected to sequencing and showed a 774-bp fragment containing the variable region of the deduced gene, domain IIS1-S4, and upstream of the 5′ extracellular intersegment linker (Fig. 3). Nucleotide sequences of the studied fragments were submitted and are accessible in the NCBI GenBank with the accession numbers MK673309-MK673335 and ON237564-ON237579. Nucleotide analysis of 47 sequences showed that 43 samples (91.6%) had three mutations (M815I, T917I and L920F), two samples (4.2%) had T917I mutation and two (4.2%) samples were wild type.

**Fig. 4:**
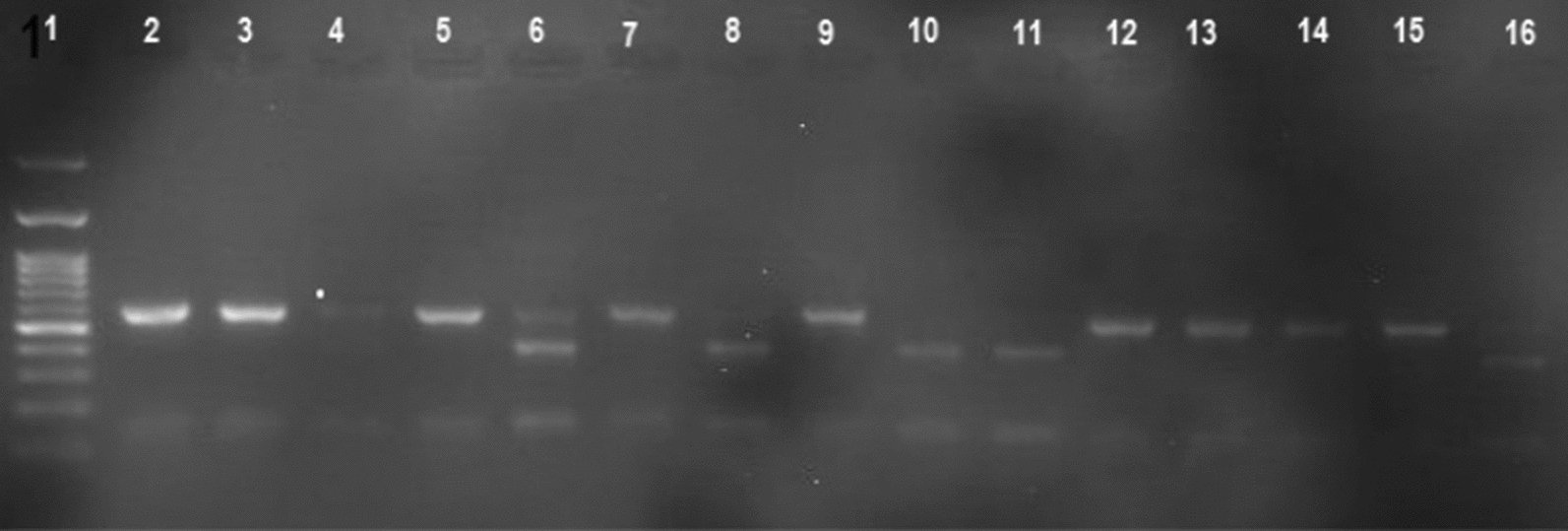
Agarose gel electrophoretic graph of the nested PCR products in head louse mitochondrial groups. Lanes: 1, 100-bp ladder; 2, 3, 5, 7, 9, 12–15, samples of clade B; 4, 6, 8, 10, 11, 16, samples of clade A

Nested PCR conducted on 300 randomly selected samples successfully amplified the fragments of mitochondrial *cytb* (Fig. 4). Amplified fragments were 100 and 470 bp in size. Among the studied samples, clade B was presented as the predominant mitochondrial group and comprised 89% of the studied populations.

**Fig. 5:**
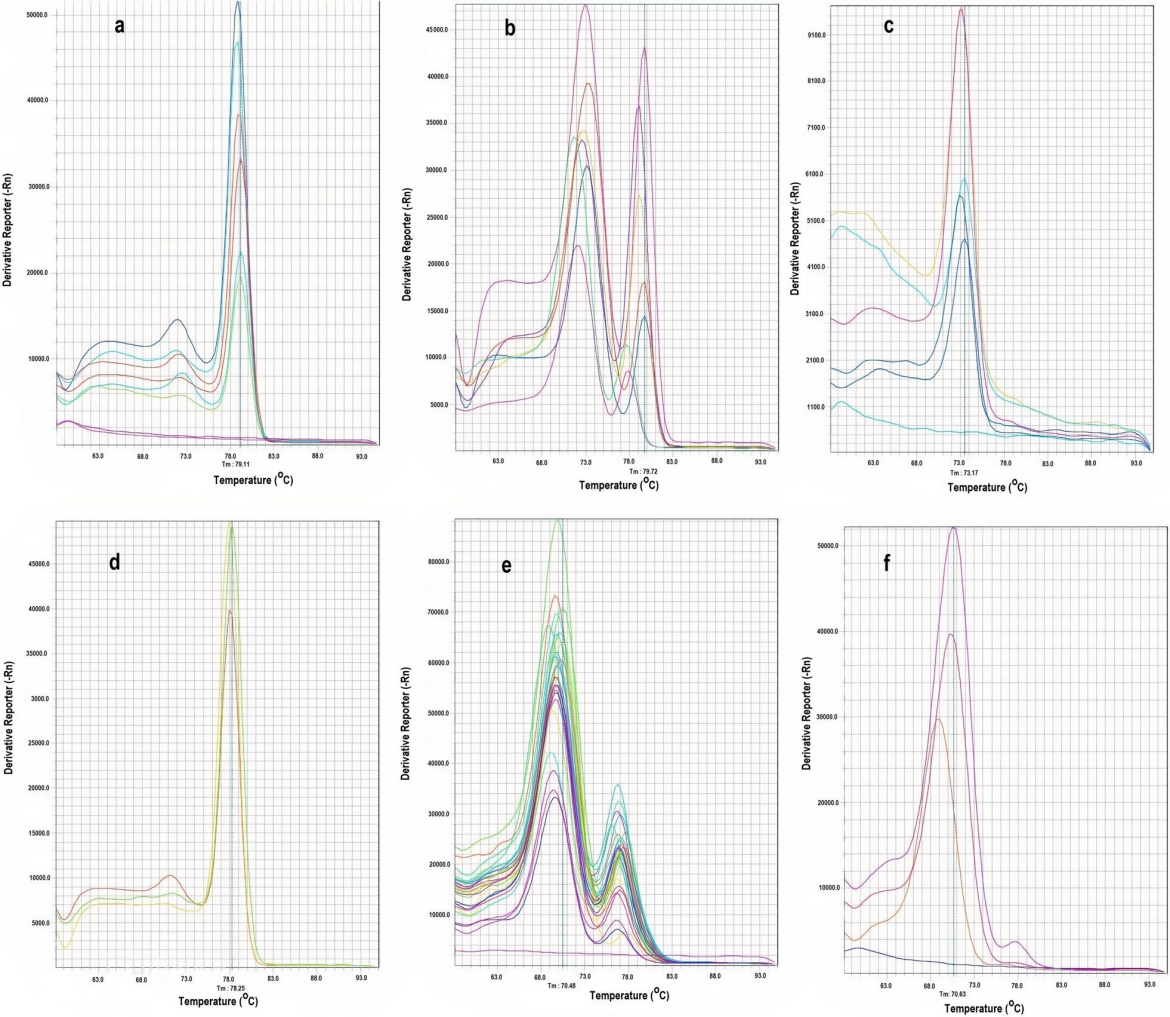
Melting curves of the two hot spot knockdown resistance region fragments in different head louse genotypic groups. **a**, **b**, **c** Fragments with M815 locus, **d**, **e**, **f** fragments with T917 and L920 loci.** a**,** d** Curves for homozygous mutant alleles,** b**,** e** curves for heterozygous alleles,** c**,** f** curves for homozygous wild alleles

SYBER Green RT-PCR amplification of domain IIS1-S3 and IIS4-S5 fragments was performed on 296 samples. Melting curve analyses of the domain IIS4-S5 amplicons displayed a single peak at 70.0 °C and 82.0 °C in homozygote mutant (I917/I917 and F920/F920) genotypes and wild-type (T917/T917 and L920/L920) genotypes that included 4.73% and 1.35% of samples, respectively (Fig. 5). There was no significant difference in the frequency of heterozygotes, which included 93.92% of the samples in clades A and B (*P* = 0.41). Processing of the melting curves of the domain IIS1-S3 fragments presented a single peak at 72.0 °C and 78.0 °C in the mutant (I815/I815) and wild-type (M815/M815) homozygotes, respectively (Fig. 5). The I815/I815, I815/M815 and M815/M815 genotypes were present in 23.31%, 67.23% and 9.46% of the studied samples, respectively (Table 2). Globally, a resistant allele frequency of 56.59% was observed in the amplicons of domain IIS1-S3. Both mitochondrial groups A and B possessed mutant alleles at a medium frequency of 48.61% and 58.69%, respectively, but their frequency was significant different (*P*_=_0.09). The heterozygote frequency in these clades was also statistically significantly different (*P* < 0.0001). The rate of resistant alleles and heterozygote frequency was higher in clade B than in clade A. The exact test for the Hardy–Weinberg equilibrium for clade B indicated that genotype frequencies significantly differed from expectation (*P* = 0.00). In addition, populations of this clade had an inbreeding coefficient (*F*_is_) < 0, suggesting an excess of heterozygotes and negative inbreeding between individuals (Table 2).

## Discussion

To the best of our knowledge, this is the first study to analyze permethrin resistance in head louse populations in the Middle East. We detected a low mortality in louse samples following exposure to permethrin by contact and topical methods. This result demonstrates a resistance to permethrin and the failure of this compound to control head lice in the studied areas. The emergence of permethrin resistance in head lice has a long history and dates back to the 1980s [49, 61]. In a related study on head louse populations in Israel, which at the time of the study had been subjected to the pressure of permethrin for 4 years, a slight decrease was observed in the LT_90_ value whenever the applied dose of permethrin increased from 0.25% to 1%. The LT_50_ and LT_90_ values for 1% permethrin in these populations were detected at 311 and 736 min following exposure, respectively [48]. Similar results were also reported in a study from the USA, with all louse populations surviving significantly longer than the susceptible reference strains [49]. A mortality analysis found that permethrin resistance was widespread in the USA, with LT_50_ and LT_90_ values of 9–16 and 12–30 h, respectively [51]. The exposure of resistant head lice to the maximum permethrin concentration (90%) generated 5–13.3% mortality in lice from Buenos Aires [50]. In the study of Hunter et al. in Australia [52], the reference strain of body louse was highly sensitive to permethrin, and 100% mortality was recorded at 15 min after exposure; however, this condition did not develop in resistant populations until after 3 h. A low efficiency of 1% permethrin against head louse populations in East Jakarta has also been recently documented [62]. The results of the present study showed 36.7% mortality at 60 min, and the LT_50_ and LT_90_ values were 37.66 and 354.6 min, respectively. These LT values are lower than those reported from surveys conducted across Europe (i.e. 83% in the UK and 14.4% in Denmark), but they are in accordance with those reported from urban areas of France [55]. Comparison of all these results is difficult since variation in mortality rate across different surveys may be due to varied application methods. However, bioassay results are strong evidence for the existence of pyrethroid resistance in the studied areas.

Studies involving topical application of permethrin to susceptible strains of human body louse reported that the LD_50_ value (lethal dose that kills 50% of exposed specimens) was 2.4 ng permethrin per louse. This value among the three permethrin-resistant Argentinian populations ranged from 40 to 643 ng/insect [57]. In contrast, the LD_50_ value for the susceptible strain of body louse was reported in the range of 0.17–0.19 ng per louse in Denmark. In this study, the discriminating dose of 2 ng permethrin per louse was reported for determining the susceptible and resistant specimens [56]. Permethrin resistance has also been documented by topical application of 2 ng permethrin in head louse populations of Buenos Aires [53]. In the present study, we used a discrimination dose (2 ng/ louse), which achieved 25% mortality and found that increasing the dose (16 ng/louse) had only a minor effect on mortality. This result is proof of the presence of resistance in the studied populations.

The bioassay results of the present study indicated non-synergistic effects of PBO on permethrin. This result agrees with those of Hemingway et al. [49] who found a weak synergistic effect of PBO on permethrin for the control of pediculosis in the first decade of permethrin application, in the time when the selection pressure of insecticide to head louse populations was low. The weak synergistic effects of PBO have also been reported from populations in South Florida, Panama and Ecuador where permethrin-resistant alleles are unstable and have a low frequency [22, 54]. In accordance with the present results, data from some reports have shown that the mortality and knockdown responses of highly resistant head lice to permethrin are significantly increased through synergy by PBO [22, 50, 54–56, 63, 64]. The authors of those studies suggest that PBO, which acts as a mixed-function oxidase (MFO) enzyme inhibitor, plays an inductive role in the suppression of cytochrome P450 activity in metabolic resistance. The permethrin-PBO combination also decreased the LC_50_ (lethal concentration that kills 50% of exposed specimens) value in highly resistant head louse populations from Argentina [50]. Despite the non-synergistic effect of PBO in the studied populations, increased permethrin selective pressure, stabilization of resistant alleles and MFO detoxifying mechanism along with *kdr* nerve insensitivity could contribute to the emergence of highly resistant populations. Taking into account the evidence of resistance and the probability of emerging highly resistant populations through the selective pressure of current control measures, it is necessary to develop resistance management programs in the studied areas and neighboring regions.

The majority of published works on insecticide resistance in head lice are largely anecdotal and non-replicated. This problem stems from the lack of laboratory colonies necessary to standardize bioassay protocols [65]. While the experimental development of head lice specimens has been reported in the last few years [66], susceptible and resistant laboratory strains of this louse have not yet been identified. Moreover, the sensitive body lice strains used in recent studies have exhibited experimental results that differ from the level of sensitivity to permethrin, which has challenged the choice of diagnostic dose and comparison of research results. The absence of standard susceptible strains in the present study makes it difficult to compare the results of various studies. Nevertheless, lower mortality or lower knockdown responses to permethrin, which were observed in the bioassay for resistance, may not correlate with the clinical failure of pyrethroids. In clinical practice, long exposure times could result in a relatively satisfactory efficiency, even on resistant lice. Further clinical trials are required to identify all contributions to pyrethroid resistance, such as head lice issues, possible host factors and alternative therapeutic measures. Likewise, the supply of effective therapeutic compounds, preparation of various health brochures to promote good health practices and long-term surveillance of resistance are important topics that should be considered by the Ministry of Health and Medical Education in Iran in the context of achieving control of pediculosis.

Three known *kdr* mutations (M815I, T917I, and L920F) were detected in the studied populations by molecular methods. This finding, which supports the data from the bioassays, indicates phenotypic and genotypic agreement of the studied populations. This matching is confirmed by previous original findings [22, 51, 54–56] that *kdr* mutations are highly associated with permethrin resistance in field populations of head lice. This situation demonstrates the insufficiency of permethrin for successful treatment of people with head lice and, ultimately, the failure of current pediculosis control programs. When control fails, the usual strategy is to use different insecticides, preferably of another class, for the next course of treatment [67]. Unfortunately, in Iran, there is no alternative pediculicide with an active ingredient other than permethrin. This gap raises the question of whether permethrin-based insecticides should be replaced by alternatives to maintain and restore louse susceptibility to pyrethroids.

In the present study, nucleic acid sequence analysis of the *vssc* gene revealed that 96% of the samples had the M815I, T917I and L920F mutations. Previous studies have demonstrated that these mutations are associated with permethrin resistance (*kdr*) in the *vssc* of human head lice [22, 31–33, 38, 51, 54–56, 68, 69]. The belief that M815I and L920F mutations coexist with the T917I mutation has led to the selection of two hotspot regions of *vssc* gene that possess M815, T917 and L920 sites, with the aim to express the frequency of *kdr* resistance alleles among head louse populations, both in the majority of earlier published studies [58], as well as in the present study. To our knowledge, the present study is the first to analyze permethrin resistance status of *kdr*-type mutation in head louse populations in Iran. The frequency of the *kdr* mutation alleles was 51.68%, with a total mean of 93.85% for heterozygous alleles. A world map of head louse populations from North and South America, Asia, the European Union, Oceania and Africa showed an overall resistance allele frequency ranging between 15% and 100% [24, 70]. This frequency is evidence that geographical variability is highly fluctuating, as it is exclusively dependent on the selection pressure using permethrin pediculicides; specifically, the head louse populations of Iran are currently under active selection pressure. The results of studies performed in France, Argentina, the USA, Israel, England, Denmark and Russia demonstrate that continuous insecticide selection pressure has increased the frequency of resistant alleles and stabilized resistance in various head lice populations [22, 31, 32, 51, 54, 55, 70]. Therefore, based on the evolutionary pattern found in developed countries, if the selection pressure of pediculicides is kept on Iranian head louse populations, the frequency of *kdr* mutations will expand, and resistance to pyrethroids will stabilize in the populations.

In the present study, the results of the molecular survey showed a high frequency of heterozygote *kdr* alleles in the studied areas. The observed excess of heterozygotes in the present study likely depended on whether or not the *kdr* mutation had an effect on individual fitness. In the fitness cost phenomenon, the heterozygous *kdr* allele in neural networks may enhance some physiological functions or traits, such as mobility, survival, fecundity, host preference, mating competition, developmental time, perception of stimuli and detection of olfactory signals [71–73]. Hence, lice harboring only one copy of the *kdr* mutation may have a higher chance to survive in a selective environment compared to those carrying two copies of the mutant alleles. The second and the most conservative option is that the *kdr* mutation has little or no effect on the fitness of individuals, resulting in persistent or slow returning of resistant alleles to susceptible states. Although the effect of fitness cost on the increasing frequency of heterozygotes has been determined in several disease vectors, we suggest that future studies should determine this relationship in head louse populations.

Genotypic analysis of the *kdr*-M815 allele in the present study detected a significant difference in inbreeding rate among mitochondrial groups, and a high *F*_is_ value in Hardy–Weinberg equilibrium of clade A samples demonstrated the presence of inbreeding in this group. Negative inbreeding in clade B samples may be related to the fitness cost effect of the *kdr* mutation. Interestingly, field observations in the present study distinguished different life history traits in samples of mitochondrial groups. Lice samples of clad B were small and more motile, and people infested with lice belonging to this clade had few nits in their hair. In contrast, lice samples of clad A had a relatively large body and low motility, and infested people had more nits in their hair. The fitness cost effect is generally reported based on reproductive efforts and developmental traits; however, little information is available on its effect on longevity and body size. The most striking results on the correlation of the *kdr* mutation with fitness cost have been reported from studies in *Aedes aegypti, Culex pipiens* and *Anopheles gambiae* [74–79]. In these reports, *kdr* insects presented an increase in locomotor and biting activities, as well as a decrease in the size of females and the number of eggs. These studies support our conclusion that various *kdr* alleles and combinations of different alleles have varied fitness costs. Further studies on sympatric mitochondrial groups are needed to discern the role of the genetic structure in developing and shaping these traits in deduced clades. We propose that the conventional and simple PCR method used in the present study to discriminate mitochondrial groups correctly be used for further studies to clarify new facts about head louse clades in the old continent. Considering that the target site insensitivity resulting from a *kdr* mutation is the main mechanism of pyrethroid resistance in the head louse population, detection of the intensity and frequency of resistant alleles is essential to the development, monitoring and evaluation of pediculosis control programs. The conventional molecular and bioassay methods used in the current study have been demonstrated to be reliable, economically feasible and reproducible, and they could precisely identify mutations in each head louse. Therefore, these methods, which are able to detect resistance early, are highly recommended to be used in the surveillance of pediculosis in different situations. They also provide a great deal of information on the biology of this louse.

## Conclusion

The findings of the present study indicate the existence of resistance to permethrin in head louse populations in northwestern Iran. The maintenance of current control measures could establish resistant *kdr* alleles and enhance the resistance level; therefore, it is necessary to develop resistance management programs in the studied areas. The information presented herein shows an enhanced frequency of *kdr* heterozygous alleles among head louse populations. Further studies are required to identify all contributions to pyrethroid resistance, such as head lice issues, possible host factors and alternative therapeutic measures.

## Data Availability

Data supporting the conclusions of this study are included within the manuscript and nucleotide sequences of the studied fragments are accessible in GenBank with the accession nos MK673309-MK673335 and ON237564-ON237579. If further clarification is needed, requests may be directed to the corresponding author.

## Abbreviations

CI: Confidence interval
*cytb*: Mitochondrial cytochrome *b* gene
DDT: Dichlorodiphenyltrichloroethane
gDNA: Genomic DNA
*kdr*: Knockdown resistance
LC_50_: Lethal concentration that kills 50% of exposed specimens
LD_50_: Lethal dose that kills 50% of exposed specimens
LT_50_: Median lethal time (time required to kill 50% of exposed samples)
LT_90_: Time required to kill 90% of exposed samples
MFO: Mixed-function oxidase
OC: Organochlorine
OTC: Over-the-counter
PBO: Piperonyl butoxide
RT-PCR: Real-time PCR
S: Segment
SR: Synergistic ratio
VSSC: Voltage-sensitive sodium channels protein
*vssc*: Voltage-sensitive sodium channel gene
